# Cryopreservation of 13 Commercial *Cannabis sativa* Genotypes Using In Vitro Nodal Explants

**DOI:** 10.3390/plants10091794

**Published:** 2021-08-28

**Authors:** Cassandra D. Downey, Gregory Golenia, Ekaterina A. Boudko, Andrew Maxwell P. Jones

**Affiliations:** 1 Canopy Growth Corporation, Smiths Falls, ON K7A 0A8, Canada; cassandra.downey@canopygrowth.com (C.D.D.); gregorygolenia@gmail.com (G.G.); kboudko@gmail.com (E.A.B.); 2 Gosling Research Institute for Plant Preservation, Department of Plant Agriculture, University of Guelph, Guelph, ON N1G 2W1, Canada

**Keywords:** *Cannabis sativa*, germplasm preservation, droplet vitrification, conventional vitrification, tissue culture

## Abstract

Cannabis has developed into a multi-billion-dollar industry that relies on clonal propagation of elite genetics with desirable agronomic and chemical phenotypes. While the goal of clonal propagation is to produce genetically uniform plants, somatic mutations can accumulate during growth and compromise long-term genetic fidelity. Cryopreservation is a process in which tissues are stored at cryogenic temperatures, halting cell division and metabolic processes to facilitate high fidelity germplasm preservation. In this study, a series of experiments were conducted to optimize various stages of cryopreservation and develop a protocol for long-term germplasm storage of *Cannabis sativa*. The resulting protocol uses a standard vitrification procedure to cryopreserve nodal explants from in vitro shoots as follows: nodes were cultured for 17 h in a pre-culture solution (PCS), followed by a 20-min treatment in a loading solution (LS), and a 60 min incubation in plant vitrification solution 2 (PVS2). The nodes were then flash frozen in liquid nitrogen, re-warmed in an unloading solution at 40 °C, and cultured on basal MS culture medium in the dark for 5 days followed by transfer to standard culture conditions. This protocol was tested across 13 genotypes to assess the genotypic variability. The protocol was successful across all 13 genotypes, but significant variation was observed in tissue survival (43.3–80%) and regrowth of shoots (26.7–66.7%). Plants grown from cryopreserved samples were morphologically and chemically similar to control plants for most major traits, but some differences were observed in the minor cannabinoid and terpene profiles. While further improvements are likely possible, this study provides a functional cryopreservation system that works across multiple commercial genotypes for long-term germplasm preservation.

## 1. Introduction

A major challenge facing the development and application of plant based medicines, including cannabis, is producing material with a consistent and well described chemical profile to ensure predictable and reproducible biological effects [1]. The medicinal and recreational properties of cannabis are largely related to the presence of cannabinoids, with the most naturally abundant being delta-9-tetrahydrocannabinolic acid (THCA), a precursor to the psychoactive compound delta-9-hydrocannabinol (THC), and cannabidiolic acid (CBDA), a precursor to the non-psychoactive but medicinally important compound cannabidiol (CBD) [2]. While cannabis is largely described and marketed based upon these two compounds, it produces a diverse array of potentially bioactive molecules and the use of single marker compounds to assess quality and uniformity of herbal products is problematic [1]. To date, at least 125 unique cannabinoids have been identified from cannabis and their biological activities and interactions are largely unknown [2]. Cannabis also produces a wide range of other compounds such as terpenes, which are responsible for the flavor and aroma profile of the product and are thought to interact with cannabinoids to modify their biological activity [3,4]. This complexity introduces a significant challenge in producing uniform products, which is required to ensure consistent medicinal activity and product quality. While the challenge of chemical standardization is not unique to cannabis, most governments have imposed strict quality assurance regulations that require careful attention to product quality and uniformity.

The final chemical composition of a plant is a product of both environment and genetics, and steps need to be taken to address both aspects to ensure reproducible results. To ensure a consistent environment, most drug-type cannabis cultivated for dried flowers or whole plant extracts are cultivated in a controlled environment such as a greenhouse or an indoor production facility. This provides a high degree of environmental control to maximize quality and ensure consistency within and among batches. The second component is to minimize genetic variability within the crop. Given the degree of variability in seed-based populations of existing drug-type cannabis [5], elite accessions are typically selected and propagated using clonal methods such as stem cuttings [6,7], or micropropagation [8]. In theory, clonal propagation should result in genetically uniform propagules and when combined with high fidelity environmental control, should permit the consistent production of uniform plant material.

Despite the combined use of controlled environments and clonal propagation, cannabis producers have anecdotally observed changes over time with clonal lines losing vigour and producing Lower levels of cannabinoids than the original plant. While this phenomenon has not been thoroughly evaluated in cannabis, a theory known as Muller’s ratchet [9,10] postulates that clonally reproducing organisms accumulate random mutations over time that leads to a decline in fitness and vigour. A recent study using whole genome sequencing identified a relatively high degree of genetic diversity within a single cannabis plant, with thousands of genetic differences identified among three samples taken from the bottom, middle, and top of the plant [11]. Intra-plant genetic variation is well documented in other species and similar to longer-lived perennials, the genetic distance increased from the bottom to the top of the plant demonstrating an accumulation of mutations with plant growth [12]. This suggests that somatic mutations are continually accumulating with plant growth, which is consistent with Muller’s ratchet and could contribute to long-term decline of clonal cannabis lines.

Given that cannabis appears to be prone to accumulating somatic mutations during regular growth and there are anecdotal reports of plant decline, it is important to develop methods to preserve the genetic integrity of elite genetics. While chemical and molecular analysis of micropropagated plants have not identified changes during micropropagation [13], this was based on short-term micropropagation and the use of inter simple sequence repeat (ISSR) markers to assess genetic fidelity. Since the accumulation of mutations is a continual process, short-term studies are not well suited to detect them. Furthermore, while ISSR markers are a useful tool for some applications, they are low resolution and likely to miss many mutations that could have occurred. In general, micropropagation often results in increased mutation rates [14] and given the intra-plant genetic diversity observed within a single mother plant [11], it is likely that micropropagation is not suitable for long term genetic preservation of cannabis.

Somatic mutations occur primarily during cell division [15], a necessary process for any plant growth system. The only way prevent mutations is by arresting the cell division process, which can be accomplished through cryopreservation. In this process, plant tissues are stored at cryogenic temperatures and can be maintained indefinitely without any cell division or metabolic activity [16]. With a suitable protocol, whole plants that are genetically equivalent to the original tissue can be produced. While the initial costs of cryopreservation can be higher than other approaches, it is often more cost effective for long term preservation and ensures that the genetic profile remains consistent during the storage period (there is potential for mutations during the regrowth phase) [17]. Developing this technology for cannabis is an important step to facilitate cost effective, high fidelity preservation of elite genetics.

To date, cryopreservation methods have been established for cannabis using suspension cultures [18], axillary meristem [19], as well as apical shoot tips [20]. While cryopreservation of suspension cultures has value for some biotechnological applications, pre-established meristems are better suited for germplasm conservation to reduce mutations and simplify the recovery process. This has been reported using in vitro shoot tips [19] and axillary meristems from whole plants [18], but was only tested with three genotypes and did not include any high cannabinoid genotypes (maximum of about 8% THC or CBD) representative of commercially relevant genetics. Given the documented genotypic variability in response to in vitro protocols and challenges in reproducibility [21,22], more work is needed to evaluate cryopreservation techniques in a broader range of commercially relevant genotypes. The objective of this project was to establish an efficient cryopreservation system, evaluate it across a diverse group of commercial genotypes, and compare the performance of cryopreserved plants to their controls.

## 2. Materials and Methods

This manuscript reports several experiments that were conducted to develop a robust cryopreservation protocol for the long-term preservation of *Cannabis sativa* germplasm, a validation experiment to test the protocol across 13 genotypes, and a comparison of plant performance between cryopreserved material vs. non-cryopreserved plants. It should be noted that the experiments were conducted using the material that was available to the researchers at the time based on production schedules and other factors. As a result, some experiments were conducted with single genotypes while others were conducted with multiple genotypes, some the genotypes used for various steps were not always the same, and some steps were conducted in parallel rather than sequentially.

### 2.1. Plant Material

*C. sativa* donor plants were cultivated indoor at Tweed Inc., Smiths Falls, ON, CAN (Canopy Growth Corporation). The genotypes selected for this study were owned by Canopy Growth Corporation and encompassed a range of phenotypes, chemotypes (historical major cannabinoid profiles are included in Supplemental Table S1), and responses to tissue culture conditions.

#### 2.1.1. Explant Preparation and Surface Sterilization

**Figure 1:**
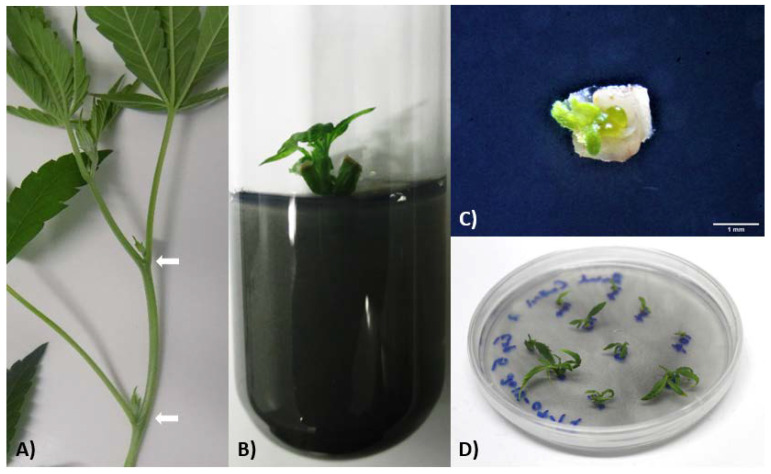
Visual representation of the micropropagation process conducted for *Cannabis sativa* in this study. (**A**) Stem cutting taken from a mother plant. Nodal segments were excised and used as starting material for micropropagation. (**B**) Surface-sterilized, 1-cm long nodal cutting inoculated on shoot initiation medium (one week since initiation). (**C**) Close-up of a micropropagated nodal segment recovering on recovery medium (one week since plating). (**D**) Nodal explants excised from four-week-old micropropagated plants and plated directly onto recovery medium (approximately four weeks since plating). White arrows denote axillary nodes located on mother plant cutting. Scale bar represents 1 mm.

Mother plants were visually inspected for pre-flower formation, disease, and general health before tissue harvest. Cuttings were taken from young stems containing a shoot tip and one or more nodes (see Figure 1A). Nodal segments (containing axillary buds), approximately 1 cm in size, were excised from each cutting with one straight cut 0.5 cm above the node, and another 0.5 cm below the node on a 45° angle. Explants were surface sterilized by full immersion in a 10% (8.25% *v/v* NaClO) Great Value™ commercial bleach (Walmart, Bentonville, AR, USA) solution supplemented with 0.1% Tween-20 (Anachemia Canada Inc., Winnipeg, MB, CAN) for 10 min (accompanied by periodic, gentle agitation). Explants were then rinsed by agitating in sterile, distilled water three times for a total of 15 min. Subsequently, explants were dried briefly on sterile, ashless Whatman™ filter paper (Cytiva, Vancouver, BC, CAN) to remove excess moisture.

#### 2.1.2. Donor Plant Initiation

Surface sterilized nodal explants were inoculated in culture tubes containing Shoot initiation medium (SIM; see Figure 1B), comprised of 4.33 g L^−1^ Murashige and Skoog (MS) basal salts and vitamins [23] (Caisson Labs, Smithfield, UT, USA), 30 g L^−1^ sucrose (Sigma-Aldrich Canada Co., Oakville, ON, CAN), 0.3 g L^−1^ activated charcoal (Anachemia Canada Inc., Winnipeg, MB, CAN), 1.86 μM kinetin (KIN), 0.53 μM naphthaleneacetic acid (NAA) (Phytotech Labs Inc., Lenexa, KS, USA), 1 mL l^−1^ Plant Preservative Mixture (PPM) (Plant Cell Technology, Washington, DC, USA), and the pH was adjusted to 5.7 ± 0.2 using 1 N NaOH and/or HCl (Fisher Scientific Company, Ottawa, ON, CAN) before adding 8 g L^−1^ agar (Caisson Labs, Smithfield, UT, USA). Fifteen milliliters of SIM was aliquoted into 25 × 150 mm borosilicate culture tubes (VWR™, Radnor, PA, USA) after heating with agitation. The vessels were then sterilized by autoclaving at 121 °C and 17 psi for 18 min.

#### 2.1.3. Donor Plant Maintenance

After at least 25 days of growth, donor plants on SIM were either used for cryopreservation immediately or transferred to fresh shoot multiplication media (SMM) for culture maintenance (see Figure 1C,D for micropropagated nodal explant growth). Shoot multiplication medium (SMM) was the same composition as SIM, excluding PPM. Generally, 50 mL of SMM was aliquoted into Magenta™ (77 × 77 × 97 mm; Sigma-Aldrich Canada Co., Oakville, ON, CAN) or Caisson^®^ GA-7 (77 × 77 × 102 mm; Caisson Labs, Smithfield, UT, USA) vessels after heating and agitation. The vessels were sterilized by autoclaving at 121 °C and 17 psi for 18 min. Cultures were maintained under a 16/8-h light/dark photoperiod at 24 ± 2 °C. Light was provided by either cool white fluorescent bulbs or LEDs (Valoya, Helsinki, Finland) and emitted a photon flux of 42–52 µmol/m^2^/s.

### 2.2. Cryopreservation Protocol

#### 2.2.1. Conditioning

Explants were placed in preculture solution (PCS) dispensed in 62 × 95 mm baby food jars (hereafter referred to as “jars”) (Phytotech Labs Inc., Lenexa, KS, USA). PCS consisted of full-strength MS basal salts (Sigma-Aldrich Canada Co., Oakville, ON, CAN) and 0.5 M sucrose. PCS was filter-sterilized using a 0.20 µm polyethersulfone membrane sterilization unit (VWR™, Radnor, PA, USA). Explants were incubated for approximately 17 h under standard tissue culture room conditions while being agitated at 155 rpm on an orbital mini shaker (VWR™, Radnor, PA, USA).

Following the initial incubation period, explants were collected in a sterile, 40 µm nylon mesh cell strainer (VWR™, Radnor, PA, USA) while the PCS was allowed to flow through and be discarded. Using sterile forceps, explants were transferred back into their original jar or to a pre-sterile, 3 mL Neptune^®^ polypropylene cryogenic vial (hereafter referred to as “vial”) (Neptune Scientific, San Diego, CA, USA). Loading solution (LS) was then added to the jar or vial at approximately 50- and 2-mL volumes, respectively The composition of LS was full-strength MS basal salts, 0.5 M sucrose, and 1.9 M glycerol (≥99.5% purity; Sigma-Aldrich Canada Co., Oakville, ON, CAN), filter-sterilized as previously described. Explants were incubated in LS for 20 min while being agitated at 155 rpm.

#### 2.2.2. Vitrification

Nodal explants from position 2–4 were used to compare standard vitrification in pre-sterilized cryogenic vials to droplet vitrification. This initial comparison was conducted using plant vitrification solution 3 (PVS3), while later experiments were done using standard vitrification to compare PVS3 with plant vitrification solution 2 (PVS2) after it was identified to be more suitable.

#### 2.2.3. Vitrification—Droplet

**Figure 2:**
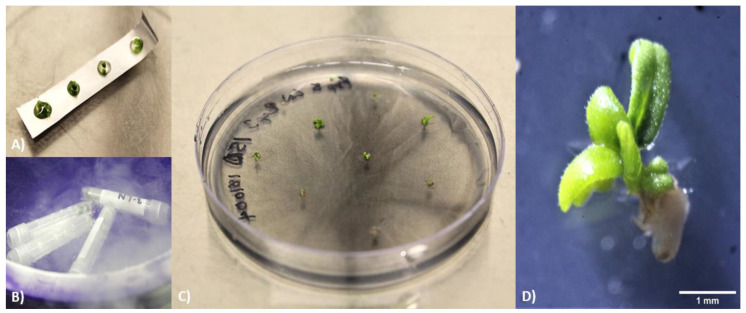
Freezing methods and recovery of cryopreserved samples. (**A**) Droplet vitrification and (**B**) vitrification performed using cryogenic vials. (**C**) Recovery of LN2 samples after four weeks on recovery medium. (**D**) Close-up of LN2 explant after four weeks on recovery medium. Scale bar represents 1 mm.

Following incubation in loading solution, explants were placed back into their original jar and submersed in approximately 50 mL of plant vitrification solution 3 (PVS3). Incubation in PVS3 occurred for 40 min with shaking at 155 rpm. While explants were incubating, autoclaved aluminum foil strips (hereafter referred to as ‘strips’) (approximately 0.5 × 2 cm) were aseptically prepared on 100 × 15 mm borosilicate glass petri dishes (VWR™, Radnor, PA, USA). Using a 5 mL polyethylene transfer pipette (VWR™, Radnor, PA, USA), single droplets of PVS3 were placed on the dull side of the strip (five drops per strip). Once incubation was complete, individual explants were placed into each of the droplets (see Figure 2A). Strips loaded with PVS3 and explants were then plunged into LN at a slight downward angle. Strips were held in the LN for a few seconds to ensure that the entirety of the unit was sufficiently frozen and then remained in LN for at least 40 min.

Approximately 25 mL of US was dispensed into sterile jars and brought to 40 °C in a hot water bath (VWR™, Radnor, PA, USA). After at least 40 min in LN, the strips containing explants were transferred into 40 °C unloading solution (US) for 30 s. US contained full-strength MS basal salts, 0.8 M sucrose, and was filter-sterilized as described previously. The jar was swirled to release the explants from the strip, and the strip was removed immediately after the explants were freed. After 30 s, approximately 25 mL of room temperature US was added to the jar to bring the solution containing the explants closer to room temperature. Explants were incubated in US for 30 min on a rotary shaker at 155 rpm.

#### 2.2.4. Vitrification—Conventional

For conventional vitrification using cryogenic vials, LS was removed and replaced with 2 mL of either plant vitrification solution 3 (PVS3) (used in earlier experiments, including droplet vs. conventional trial) or PVS2 (used in later experiments after PVS2 was found to perform better). The composition of PVS3 was full-strength MS basal salts, 50% sucrose, and 50% glycerol. PVS2 included full-strength MS basal salts, 0.4 M sucrose, 30% glycerol, 15% ethylene glycol (Fisher Scientific Company, Ottawa, ON, CAN), and 15% dimethyl sulfoxide (DMSO) (Phytotech Labs Inc., Lenexa, KS, USA). Both solutions were filter-sterilized as previously described. The vials were incubated at 155 rpm for 5 min before the solution was removed and replaced with 2 mL of fresh vitrification solution (VS). From here, vials were incubated while being shaken at 155 rpm for a predetermined amount of time (20–80 min). Following this incubation period, vs. was discarded and vials were replenished with 0.5 mL of fresh VS. Vials were immediately submerged in liquid nitrogen (LN; supplied by Linde Canada Inc., Mississauga, ON, CAN) and held there for at least 30 s with forceps to ensure adequate freezing of the explants. Vials were kept in LN for at least another 40 min (see Figure 2B).

Approximately 50 mL of distilled water was dispensed into jars and brought to 40 °C in a hot water bath. After at least 40 min in the LN, vials were removed and immediately placed in jars containing the preheated water. Jars were continually swirled for 90 s to allow the explants to thaw. Subsequently, the vials were transferred to new jars containing approximately 50 mL of room temperature water. Jars were swirled over the duration of 60 s before the vials were removed. vs. was discarded from the vials and replaced with 1 mL of US. Vials were incubated for 30 min on a rotary shaker (155 RPM).

#### 2.2.5. Recovery Preparation

After the unloading step, US was removed by either passing through a cell strainer into a waste bottle (the strainer collecting explants from jars) or by use of a pipette (leaving only explants in the vials). Using sterile forceps, explants were transferred to autoclaved, ashless filter paper to blot dry. Subsequently, explants were plated onto recovery media, right-side up (or on their side if orientation could not be discerned). Please see Figure 2C for a visual representation of samples plated on recovery media.

#### 2.2.6. Recovery and Data Assessment

Samples were allowed a recovery period of 30 days in culture. Thereafter, growth was assessed and categorized in terms of survival and regeneration. ‘Survival’ was classified as explants that remained green but may not have shown visible growth. ‘Regrowth’ was demonstrated by leaf development on explants (see Figure 2D). Samples were analyzed using a stereo microscope (Leica Microsystems, Wetzlar, Germany). For all cryopreservation experiments, three treatment groups (‘control’, ‘no LN2’, ‘LN2’) were compared. ‘Control’ samples were excised from the donor plant and plated directly onto recovery media to determine the baseline survival rate of the explants. ‘No LN2’ samples were exposed to the cryopreservation protocol but were not frozen in LN to assess the effect of pretreatments on explant health. ‘LN2’ samples were processed through the entire cryopreservation protocol including the freezing process. At least 10 explants were situated on each plate, and each cryopreservation and recovery media treatment were replicated at least two times. Contaminated plates were removed from the data when performing statistical analysis.

#### 2.2.7. Growth Conditions for Recovering Samples

Samples were incubated in the dark in a culture room (24 ± 2 °C) for a predetermined amount of time before either gradual or rapid exposure to ambient light conditions (16/8-h light/dark photoperiod) under cool white fluorescent or LED lighting as previously described.

#### 2.2.8. Experiment—Recovery Media

A variety of recovery media were investigated using cryopreserved Cultivar 1 samples. The media tested included SMM, half strength SMM (HalfSMM), which was the same as SMM except made with half-strength MS basal salts with vitamins, HalfSMM with supplemental GA_3_ (HalfSMM + GA_3_) which was composed of HalfSMM with the addition of 1 μM Alfa Aesar™ gibberellic acid (GA_3_; Fisher Scientific Company, Ottawa, ON, CAN), MS basal medium (MSbasal) composed of full-strength MS basal salts with vitamins, 30 g L^−1^ sucrose, 0.3 g L^−1^ activated charcoal, 8 g L^−1^ agar, and MSbasal with GA_3_ (MSbasal + GA_3_) composed of MSbasal supplemented with 1 μM GA_3_.

GA_3_ was prepared as a 100 mM working stock solution by dissolving the powder in 99% ethanol before diluting it with distilled water. All media were pH adjusted to pH 5.7 ± 0.2 before the addition of agar and autoclaved before being aliquoted into vessels as described previously. Approximately 25 mL of autoclaved media was dispensed into pre-sterile, 100 × 15 mm plastic petri dishes (VWR™, Radnor, PA, USA) under aseptic conditions.

Ten explants were used per treatment and each treatment was replicated thrice. Sample response to recovery media were assessed using only the droplet vitrification protocol for SMM, HalfSMM, and HalfSMM + GA_3_, while both strip and vial protocols were performed for the recovery of Cultivar 1 explants on SMM, MSbasal, and MSbasal + GA_3_ media. All samples were cryopreserved using PVS3 with an exposure time of 40 min. The comparison of multiple genotypes responding to the cryopreservation protocol was performed only using MSbasal for the recovery media.

#### 2.2.9. Experiment—Cold Incubation of Donor Plants

The effect of cold incubation of donor plants before use for cryopreservation was investigated using Cultivar 2. Briefly, half the cultures were placed into an incubator (Norlake^®^ Tissue Culture Chamber Model; Standex International Corporation, Salem, NH, USA) programmed to 10 ± 1 °C (16/8-h day/night photoperiod, approximately 42 µmol/m^2^/s) after 4 weeks of growth. These cultures were maintained for 7 days at this temperature while the other half remained at 24 ± 2 °C. Both sets of cultures were used for cryopreservation at week 5.

Ten explants were used per treatment and each treatment was replicated thrice. Samples were cryopreserved using the conventional cryopreservation protocol and a 40-min exposure to PVS3. Samples were plated onto MSbasal recovery medium for recovery. Samples recovered under one of two light regimes: darkness for 7 days, gradual build-up to ambient light intensity for 7 days, then ambient light intensity for 16 days; darkness for 7 days, then ambient light intensity for 23 days. Light was provided by programmable LEDs. Sample survival and recovery were assessed 30 days after plating.

#### 2.2.10. Experiment—Extension of Incubation in Darkness

The response of cryopreserved Cultivar 1 samples to increased incubation in darkness during the recovery period was investigated. Ten explants were used per treatment and each treatment was replicated thrice. Samples were cryopreserved using the conventional cryopreservation protocol and a 40-min exposure to PVS3. Samples were plated onto MSbasal recovery medium for recovery.

Light treatments included the following:

(1) Darkness: 5 days, gradual light: 5 days, ambient light: 20 days
(2) Darkness: 10 days, gradual light: 5 days, ambient light: 15 days
(3) Darkness: 15 days, gradual light: 5 days, ambient light: 10 days
(4) Darkness: 20 days, gradual light: 5 days, ambient light: 5 days
(5) Darkness: 7 days, gradual light: 7 days, ambient light: 16 days
(6) Darkness: 14 days, gradual light: 7 days, ambient light: 9 days
(7) Darkness: 14 days, gradual light: 0 days, ambient light: 16 days
(8) Darkness: 21 days, gradual light: 7 days, ambient light: 2 days

During the period of gradual light exposure for treatments 1–4, five sheets of white printer paper were placed on top of the cultures. One sheet of paper was removed each day for the five-day period, which was succeeded by the samples being subjected to normal culture room light intensity. For samples treated with light treatments 5–8, the light source was programmed to slowly increase in magnitude for the duration of the gradual light intensity stage until ambient levels were achieved. Light was provided by cool white fluorescent bulbs (treatments 1–4) or programmable LEDs (treatments 5–8).

#### 2.2.11. Experiment—Comparison of PVS3 and PVS2

The recovery of Cultivar 2 samples after cryopreservation using either PVS3 or PVS2 in cryogenic vials was investigated. Ten explants were used per treatment and each treatment was replicated thrice. The preliminary exposure time to PVS3 (40 min) was compared to PVS2 exposure times of 20, 30, 40, 60, and 80 min. Samples were plated onto MSbasal recovery medium and allowed to recover under darkness for 5 days, followed by low light conditions (5 days) then ambient light (20 days). Sample survival and recovery were assessed 30 days after plating.

#### 2.2.12. Growth Conditions for Donor Plants and Recovered Cultures

**Figure 3:**
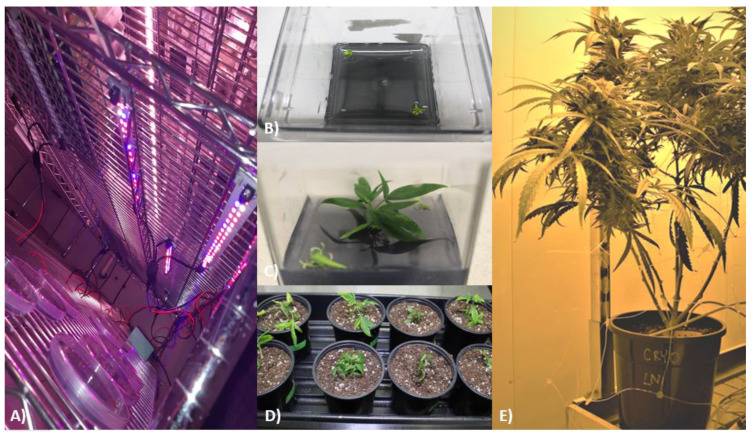
Visual representation following the recovery, ex vitro cultivation, and flower induction of *Cannabis sativa* Cultivar 1 control, no LN2, and LN2 samples. (**A**) Samples recovering from the cryopreservation process under LED lighting; (**B**) LN2 explants transferred from recovery media to SMM; (**C**) LN2 plantlet after 2 months on SMM; (**D**) control, no LN2, and LN2 plants transferred to ex vitro conditions (photo taken 1 week after transfer); (**E**) flowering LN2 plant ready for harvest.

Samples that recovered from cryopreservation (demonstrating at least new leaves) were subcultured on either SMM or SMM supplemented with 2 µM GA_3_ (SMM + GA_3_) and were allowed to grow for at least four weeks. Cultures grew under a 16/8-h light/dark photoperiod at 24 ± 2 °C. Light was provided by either cool white fluorescent bulbs or LEDs (see Figure 3A) and emitted a photon flux of 42–52 µmol/m^2^/s.

#### 2.2.13. Experiment—Cultivar Response to Cryopreservation Protocol

The optimized cryopreservation protocol was used to assess the survival and regrowth of 13 commercial cannabis genotypes. Briefly, samples were vitrified using conventional vitrification using an exposure period of 60 min in PVS2. Samples were recovered on MSbasal medium in the dark for five days followed by 25 days at 42 µmol/m^2^/s. Ten explants were used per cultivar and each cultivar was replicated thrice. Survival and recovery were assessed at 30 days post-cryopreservation.

#### 2.2.14. Evaluation of Cryopreserved Plants

A subsample of control (*n* = 4), no LN2 (*n* = 2), and LN2 (*n* = 2) Cultivar 1 cultures from early trials (droplet vitrification method, 40-min PVS3 exposure, gradual exposure to light supplied by cool-white fluorescent bulbs) were selected for hardening off and ex vitro growth (see Figure 3B,C). These samples were used for subsequent phenotyping and measurement of specific chemical compounds commonly detected in cannabis inflorescence (hereafter referred to as “bud”) and trim material. Cultures that were selected for ex vitro growth displayed visually normal morphology (explants developing elongated shoots) and had developed adventitious roots while in culture without a specific rooting treatment.

The plants were transferred out of culture on 26 June 2018. Approximately 15 mL of autoclaved tap water was poured into the vessel of each plant being transplanted and the media was allowed to soften. The plants were subsequently removed from the vessels and the roots were separated from the medium under running tap water. A small amount of tap water was added to each vessel along with their respective sample to ensure that the roots would not dry out.

Individual plants were planted in 500 mL pots, which were filled with moist, autoclaved, soilless potting mix (Pro-Mix HP^®^ Mycorrhizae, Pro-Mix, Rivière-du-Loup, QC, CAN). Briefly, the roots of the plants were placed into the small hole created in the center of each pot and covered with the potting mix (see Figure 3D). Once the plants had been transferred, they were watered with approximately 80 mL of diluted half-strength vegetative fertilizer. The pH and EC of the veg feed were approximately 6.5 and 1.0, respectively. Plants were watered with this for one week after transplanting. For the first 96 h, the plants were given 80–100 mL of feed daily. Following that, the plants were watered as needed. Beginning in the second week of hardening, the plants were watered with veg 1 solution (EC 2.0).

The potted plants were placed into a clone tray (27.8 × 54.5 × 6.2 cm; T.O. Plastics^®^, Clearwater, MN, USA), transferred to a clone propagation cart located in an environmentally controlled grow room, and covered with a humidity dome. The vents on the humidity dome were opened after 48 h, and the dome was removed 96 h after transplanting. For 7 days, the light levels were set to half of the maximum intensity (approximately 150 µmol/m^2^/s), supplied by T5 fluorescent bulbs and programmed to an 18/6-h day/night photoperiod. After the initial week, the light intensity was increased to approximately 250 µmol/m^2^/s.

#### 2.2.15. Vegetative Growth

After new growth was observed and roots were seen protruding from the sides of the pots, the plants were transferred to 3.79 l pots filled with growing medium and relocated to a grow room for vegetative growth (week of 9 July 2018). The plants were allowed to grow for approximately 2 months under an 18/6-h day/night photoperiod and light supplied by high pressure sodium (HPS) and metal halide (MH) lights. Dead and yellowing leaves and lateral stems in close proximity to the base of the plant were removed as needed to ensure adequate air flow and reduce the risk of disease. Plants were watered with pH- and EC-adjusted nutrient solution as needed via drip irrigation.

#### 2.2.16. Flower Induction

On 12 September 2018, plants were subjected to a 12/12-h light/dark photoperiod for floral induction. Light was supplied by HPS and MH ballasts. Plants were watered with pH- and EC-adjusted nutrient solution as needed via drip irrigation. The plants were subjected to a nutrient flush with water one week before harvest (see Figure 3E).

#### 2.2.17. Bud and Trim Harvest

On 7 November 2018, whole plants were cut at the base of the stem (just above the medium) and whole plant weights were taken immediately. Lateral branches were cut from the main stem and both trim and bud tissues were removed from the branches. The combined weights of the main stem and lateral branches were subsequently taken.

Fresh bud was hand-trimmed to remove any additional fan leaves and stem; these tissues were added to the trim material. Weights of freshly harvested plants, bud, and trim were recorded; fresh stem weights were calculated by subtracted fresh bud and trim weights from the total plant weight. Fresh bud and trim harvested from individual plants were then transferred to a drying room (16–21 °C, 35–65% relative humidity), spread over stainless steel screens (Bundy Baking Solutions, Urbana, OH, USA) placed on drying racks (Metro^®^ 2660 Dry Unit, Metro Shelving, Curtis Bay, MT, USA) and allowed to dry for at least 7 days.

#### 2.2.18. Moisture Analysis

Before harvested plant material could be packaged, moisture content was measured using a Mettler Toledo^®^ HE73 Moisture Analyzer (Mettler Toledo, Mississauga, ON, CAN) with a run temperature set to 95 °C. A subsample of bud weighing 3–5 g (exact weight recorded) was milled using a hand grinder onto an aluminum pan and loaded into the heating module. Sample moisture content was expressed as a percentage once drying had commenced. The harvested product could be packaged and used for further analysis once moisture content had reached <9%. Product was packaged in a plastic packaging pouch that was either heat (Uline^®^ Tabletop Poly Bag Sealer, Uline Canada, Milton, ON, CAN) or vacuum (Henkelman Vacuum Systems^®^ Boxer 42 XL, Henkelman BV, CJ ‘s-Hertogenbosch, Netherlands) sealed and subsequently stored at room temperature.

Dry weights for the bud and trim from each plant were recorded immediately before packaging and bud and trim moisture content (%) and bud yield per plant (g/g dry weight) were calculated.

#### 2.2.19. Cannabinoid and Terpene Detection and Quantification

Approximately 10 g of bud and trim sample (actual weight recorded) was submitted for measurement of cannabinoids and terpenes for each individual plant.

For cannabinoid analysis, samples were homogenized using a mortar and pestle. 0.5000 g (+5% tolerance) of milled sample was weighed and transferred to a 10 mL test tube. Extraction solution (10 mL) was added to each sample. Each sample was then vortexed for 30 s, sonicated for 30 min, then vortexed a second time. The supernatant from each sample was transferred to a dilution vial (A) using a 3- or 10-mL glass or plastic 0.2 µm filter syringe after centrifugation for 3 min at 1000× *g*. The first couple milliliters of filtrate was discarded and not used for analysis in case cannabinoids were bound to the membrane. 50 µL of sample from dilution vial A was transferred to a second dilution vial (B). 900 µL of dilution solution was added to dilution vial B along with the sample. Cannabinoids were measured using an Agilent Technologies High Performance Liquid Chromatography (HPLC)—1200 Infinity system with a diode array detector (DAD) (Agilent Technologies Inc., Santa Clara, CA, USA).

For terpene analysis, approximately 5 g of sample was homogenized in a mortar and pestle and transferred to a 15 mL plastic centrifuge tube. Approximately 500 mg (+2% tolerance) of homogenized sample was loaded into the headspace vial directly. Terpenes were measured using an Agilent 7820A/7890B gas chromatograph (GC) system with flame ionization detection (FID) (Agilent Technologies Inc., Santa Clara, CA, USA). Data obtained from the samples was analyzed by Chemstation^®^ software (Open LAB CDS Chemstation Edition Rev. A.02.02(1.3), ChemStation International Inc., Dayton, OH, USA).

A total of nine cannabinoids and 23 terpenes were investigated for this study. Peaks on the chromatographs were identified by internal (in-house partially decarboxylated bud material) and external cannabinoid (Cerilliant Corporation, Round, Texas, USA) and external terpene (Sigma-Aldrich Canada Co., Oakville, ON, CAN; Restek Corporation, Bellefonte, PA, USA) standards. Final values are provided as %w/w of the original dried material (see Supplemental Figures S1 and S2 for HPLC-DAD and GC-FID chromatographs, respectively).

### 2.3. Statistical Analysis

Data was compared using two-way ANOVA followed by Tukey’s HSD test if the model was significant. Statistical analyses were conducted using R version 4.1.0 [24]. Statistics are presented as means with standard errors, and significant differences are denoted by different lowercase letters (α = 0.05).

## 3. Results and Discussion

### 3.1. Pre- and Post-Cryopreservation Conditions

#### 3.1.1. Recovery Media

Five recovery media were investigated for their effectiveness in supporting explant survival and recovery using cryopreserved Cultivar 1 samples (see Table 1). All recovery media tested were supplemented with activated charcoal to prevent the accumulation of harmful phenolic compounds and inhibitory gases in the explants [25]. SMM media—generally used for maintenance of in vitro donor plants—was supplemented with KIN and NAA, which have been documented in inducing shoot proliferation and root induction in micropropagated samples, respectfully [26].

A half-strength MS salts formulation was included as a treatment on the assumption that normal MS nutrient concentrations had the potential to contribute additional stress on freshly cryopreserved tissues. The recovery of other plant species, such as orchid, have shown to benefit from minimalistic regeneration media after cryopreservation. For example, the regeneration of plants from cryopreserved protocorm-like bodies of *Bratonia* was successful when plated on half-strength MS medium, void of plant growth regulators [27].

GA_3_ is known for its role in shoot elongation [28], which was difficult to promote in the LN2 samples throughout this study (data not shown). The addition of this hormone into the recovery media increased the frequency of shoot elongation in no LN2 samples but induced chlorosis and hyperhydricity LN2 samples (data not shown). Therefore, GA_3_ was not included in the recovery media of subsequent experiments.

The results presented in this study showed no significant differences in sample survival or recovery in respect to the vitrification method used (conventional or droplet), and recovery media was only significant for sample recovery (not survival) when the droplet vitrification method was used. Based off of these results, subsequent experiments used MS basal medium (void of plant growth regulators) for sample recovery.

#### 3.1.2. Cold Incubation of Donor Plants

**Figure 4:**
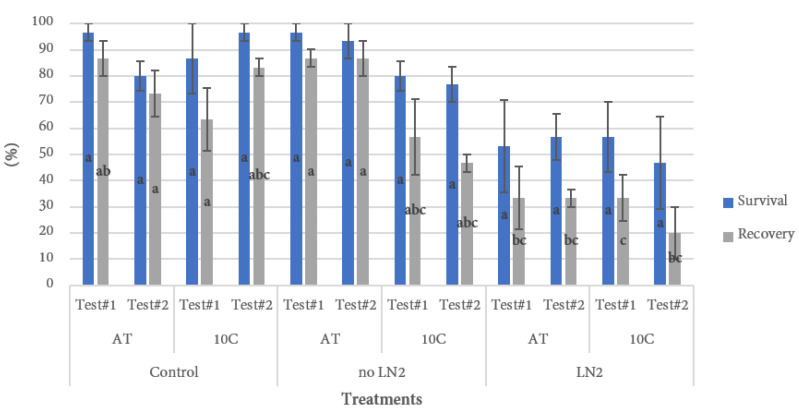
Survival and regrowth of *Cannabis sativa* Cultivar 2 explants plated on MS basal recovery medium and incubated under various light treatments. Donor plants were either grown under common tissue culture temperatures (24 ± 2 °C) or incubated under cold conditions (10 ± 2 °C) one week before explant excision. Survival and recovery were analyzed separately. Bars displaying significant differences are denoted by different lower-case letters (*p* < 0.05). AT: Ambient temperature. Test#1—Light treatment: darkness for 7 days, gradual light for 7 days, ambient light for 16 days. Test#2—Light treatment: darkness for 7 days, ambient light for 23 days.

While the process of vitrification, freezing, and thawing are critically important to develop a cryopreservation protocol, there are several factors both before and after the process that can impact the outcome. One common approach to improve survival is to expose the plants to cold conditions to harden them prior to the cryopreservation process [29,30]. This process elicits the natural adaptive mechanisms of cold tolerant plants that may include the accumulation of specific proteins, sugars, and other compounds that protect them from damage [29], and has been used to improve cryopreservation success in various species [31,32]. However, in the current study a one-week pre-cold treatment at 10 °C provided no benefit for survival, and had significantly negative consequences for recovery (Figure 4). In addition to the pre-cryopreservation conditions, the post-cryopreservation conditions can play an important role in survival and regrowth of plants. Two different lighting treatments were used for recovery: one with a slow progression from dark to ambient light conditions (over seven days), and one with a rapid change from darkness to normal light intensity. Neither light treatment had a significant impact on sample survival or regrowth.

#### 3.1.3. Extension of Incubation in Darkness

**Figure 5:**
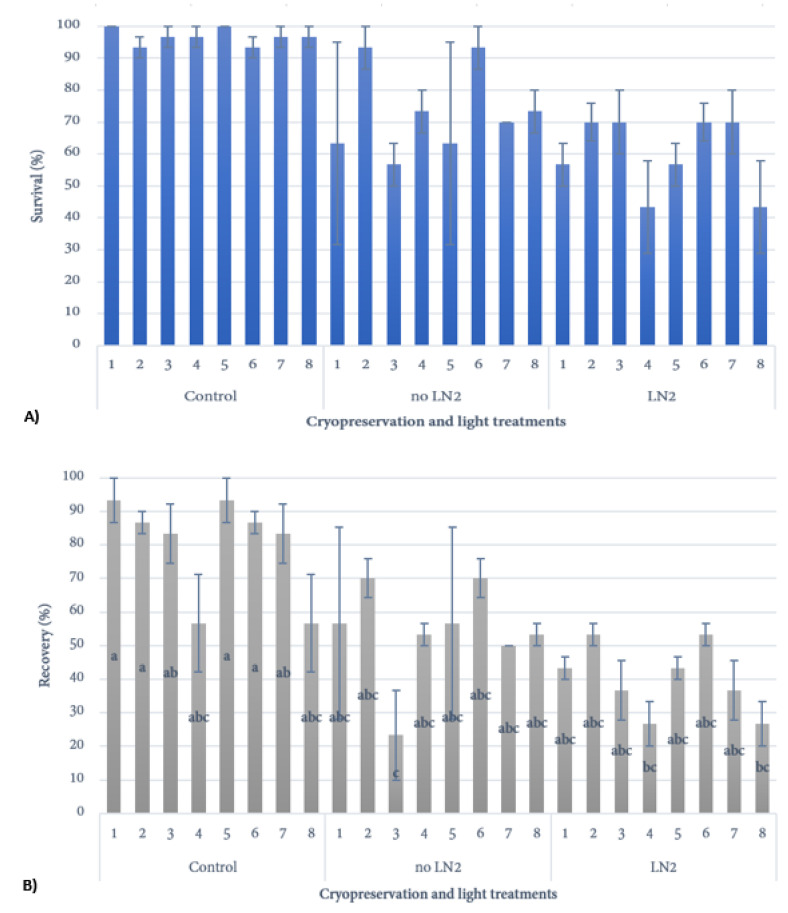
Survival (**A**) and regrowth (**B**) of *Cannabis sativa* Cultivar 1 explants plated on MS basal recovery media and incubated in various times of darkness before exposure to low and ambient light conditions. Survival and recovery were analyzed separately. Bars displaying significant differences are denoted by different lower-case letters (*p* < 0.05). Light treatments: (1) darkness: 5 days, gradual light: 5 days, ambient light: 20 days; (2) darkness: 10 days, gradual light: 5 days, ambient light: 15 days; (3) darkness: 15 days, gradual light: 5 days, ambient light: 10 days; (4) darkness: 20 days, gradual light: 5 days, ambient light: 5 days; (5) darkness: 7 days, gradual light: 7 days, ambient light: 16 day; (6) darkness: 14 days, gradual light: 7 days, ambient light: 9 days; (7) darkness: 14 days, gradual light: 0 days, ambient light: 16 days; (8) darkness: 21 days, gradual light: 7 days, ambient light: 2 days.

Another important post-cryopreservation is light quality [33]. While light plays an important role in plant growth and development, it can also represent a significant stress that can have negative consequences in a plant tissue culture setting. For example, light is known to upregulate the phenylpropanoid pathway, which can lead to the production and accumulation of toxic phenolic compounds and tissue browning [34]. A common method to reduce tissue browning or exudation is to reduce light levels [35]. In the case of cryopreservation, the tissues have been exposed to potentially phytotoxic compounds in the vitrification solution as well as extreme cold temperatures during the cryopreservation process. To mitigate these potential stresses the tissues have been exposed to, several papers have treated the explants with antioxidants to reduce oxidative stress [36] and it is common practice to include a dark period post-cryopreservation before slowly re-introducing light [8]. In the present study, there were no significant differences in the survival of samples among post-warming dark periods ranging from 5–21 days (Figure 5A), but there was a general downward trend in sample recovery (Figure 5B), suggesting that a five-day period was sufficient.

### 3.2. Duration of Vitrification Treatment

Vitrification is a process in which the accumulation of cryoprotectants within the cell encourages the aqueous components to form a metastable glass rather than crystalizing during the freezing process [37]. The reduction of ice crystallization helps to protect the cellular components from damage, thereby increasing survival rates and enabling regrowth. An important component of this process is the composition of the vitrification solution, which contain a variety of cryoprotectant compounds in different combinations and concentrations. While these compounds offer protection from freezing damage, many are phytotoxic, and the ideal solution will maximize cryoprotectant properties while minimizing phytotoxicity. This is complicated by the variable sensitivity of different species, and even tissues, to freezing damage and chemical toxicity, making it necessary to evaluate on a case-by-case basis.

**Figure 6:**
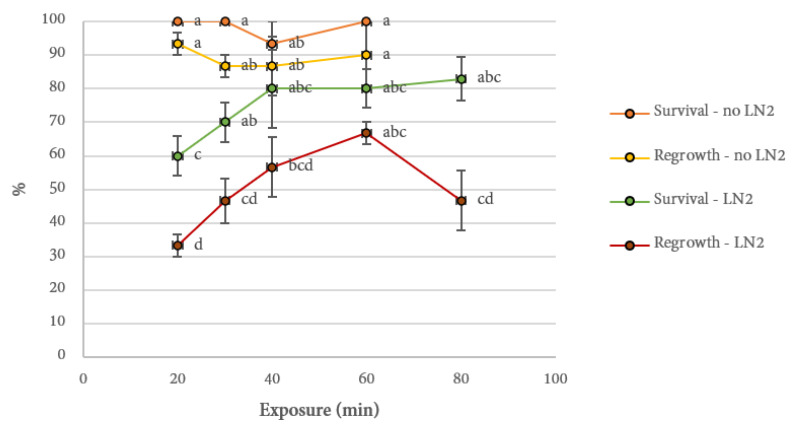
Survival and regrowth of *Cannabis sativa* Cultivar 2 explants from different exposure times in PVS2. Survival and recovery were analyzed separately. Data points displaying significant differences are denoted by different lower-case letters (*p* < 0.05).

In this study, PVS3—composed of membrane-impermeable sucrose (50% *w/v*) and glycerol (50% *w/v*)—was initially investigated for its cryoprotectant effects in *Cannabis* cryopreservation because of its potentially reduced risk of inducing phytotoxicity compared to other published plant vitrification solutions [38]. Explants exposed to the sucrose and glycerol solution would undergo a process of plasmolysis (a reduction of water content within the cell) caused by changes in osmotic pressure [39]. Volk and Caspersen (2017) found that the components of PVS3 were impermeable to the cellular membranes of *Ipomoea batatas* cell suspension cultures, suggesting that water had been drawn out of the cells, reducing the chance of ice crystal formation during freezing and thawing. In contrast, PVS2 contains both impermeable—0.4 M sucrose and 30% (*w*/*v*) glycerol—and membrane-permeable—15% (*w*/*v*) DMSO and 15% (*w*/*v*) ethylene glycol—components. The impermeable components of PVS2 serve to facilitate cell plasmolysis, while the permeable compounds contribute to deplasmolysis, an event not observed within 3 h of treating *Ipomoea batatas* cell cultures with PVS3 [40]. The differences in the mode of actions between the two vitrification solutions often influence the successful cryopreservation of different species, accessions, and explant types [39]. Initial trials were consistent with Uchendu et al. (2019) [20] in that PVS2 was more suitable than PVS3 for the cryopreservation of cannabis explants (data not shown). However, in their study they observed a toxic effect of PVS2 in the control tissues (treated with PVS2 but not exposed to LN) within 30 min of treatment and identified 15–20 min as the optimal duration. In contrast, the present study found no reduction in viability of control tissues even after a 60-min incubation and the ideal duration for explant regrowth was 60 min (Figure 6). However, as discussed above the current study used nodal explants containing axillary buds while the previous study used apical shoot tips. It is likely that the actively growing apical shoot tips are more sensitive to the chemicals present in PVS2 or absorb them quicker than the quiescent nodal explants used in this study. In another study, axillary meristems from whole plant were also successfully cryopreserved using a 20 min incubation in PVS2 [19], but the regrowth rate was substantially lower than what was observed in apical shoot tips despite using the same genotypes [18,19]. While there are many other potential contributing factors that could have led to this, it is possible that a longer incubation would have been beneficial for the nodal explants.

### 3.3. Droplet Vitrification vs. Cryo Vial Vitrification

As mentioned, vitrification is an important aspect of developing a cryopreservation protocol and is primarily accomplished using vitrification solutions (i.e., PVS2) [3]. However, another important factor is the speed in which tissues are frozen. The faster a tissue reaches the final cryogenic temperature the less chance there is for ice crystals to form during the process, so the goal of many vitrification-based cryopreservation systems is to freeze the samples as rapidly as possible.

In standard vitrification methods, the tissues are placed in a sealed cryovial and placed in liquid nitrogen to rapidly freeze the tissues. Droplet vitrification is a modification developed to expedite the freezing process by placing the tissues in a small droplet of vitrification solution on metal that is directly immersed in liquid nitrogen. The small volume in combination with the efficient heat conducting properties of the metal result in quicker tissue cooling and, in some cases, greater regrowth rates [37]. However, since many microbes and viruses can survive cryogenic temperatures, directly exposing the sterile tissues to liquid nitrogen introduces the risk of contamination. Contamination of Cultivar 1 explants exposed directly to liquid nitrogen using the droplet vitrification method was experienced in a trial conducted earlier in this study (data not shown), likely caused by microbial contaminants in the liquid nitrogen. This finding compelled the researchers to explore the possibility of performing cryopreservation using the cryogenic vial method to obtain similar recovery rates to droplet vitrification while simultaneously reducing the risk of explant contamination. As such, we compared the response of explants cryopreserved in sealed cryogenic vials vs. droplet vitrification on a variety of recovery media.

When averaged across culture media, standard vitrification and droplet vitrification had similar regeneration rates at 16.1% and 15% respectfully (Table 1; note that these trials were conducted using PVS3, resulting in relatively low recovery rates). While there were numerical differences among recovery media tested, the response was highly variable, and the differences were not significant. Due to the lack of differences between standard and droplet vitrification methods, cryogenic vials were subsequently used to simplify the process and minimize the chances of contamination.

### 3.4. Genotypic Variation

*Cannabis sativa* is known to demonstrate a high degree of genotypic variability to in vitro protocols ranging from callus growth, shoot multiplication rates, and the prevalence of physiological disorders [21]. As such, it is useful to evaluate any protocol across multiple genotypes to determine how robust a protocol is. In the present study, the final cryopreservation protocol was tested on 13 different commercial genotypes including high THC, high CBD, and mixed genotypes (see Supplemental Table S1). While the cryopreservation protocol was successful with all 13 genotypes, the regeneration rates ranged from 26.7–66.7% (Table 2). This compares to regrowth rates ranging from 57–67% in apical explant of three genotypes reported by Uchendu et al. (2019) [20], and rates of 42–44% in using axillary buds of two of the same genotypes reported by Lata et al., 2019 [19]. Overall, the regrowth rates in the present study are similar to previous reports but have a wider range as expected based on the number of genotypes that were included.

Uchenda et al. (2019) [20] reported that regrowth rates (57–67%) were relatively modest compared to the survival rates, which were up to 83%. Similarly, the survival rate in the present study (43.3–80%), was substantially greater than the regrowth (26.7–66.7%), indicating that many explants survived the freezing and thawing stage but failed to resume growth. Interestingly, this difference was not observed with axillary buds collected from whole plants, where the survival rate (45–47%) was only marginally greater than the regrowth rate (42–44%) [19].

In the present study, explants that went through all the cryopreservation steps—excluding freezing—had regeneration rates ranging from 66.7–90%, with survival rates ranging from 80–100%, indicating that much of the tissue death and stress resulted from the freezing process rather than direct phytotoxicity of the vitrification solution or other steps involved. As such, while the PVS2 solution was superior to PVS3 as reported by Uchendu et al. (2019) [20], in this study it did not provide full protection from freezing damage and improvements may be obtained by altering the composition of the vitrification solution or other aspects of the protocol.

While adjusting the vitrification solution or other aspects of the cryopreservation protocol itself may improve the outcome, the quality of starting material can also have a major impact on the success of cryopreservation. In this study, all plant material was cultured on MS based medium, which may not be ideal for *C. sativa.* A recent study found that MS based medium results in poor growth/multiplication and high degree of hyperhydricity, and that DKW based media performed better [21]. Furthermore, some genotypes performed particularly poor on MS based medium, especially over multiple subcultures, which contributes to genotypic variability. This issue is demonstrated by the variability in both survival (76.7–100%) and regrowth (30–100%) observed in the control explants that were cultured onto fresh medium without going through any of the cryopreservation steps. Based on the relatively low and variable rate of regrowth observed in the control explants, it is likely that addressing the basic culture conditions may substantially improve success with little/no direct changes to the cryopreservation protocol.

### 3.5. Evaluation of Ex Vitro Cultivated Cryopreserved Plants

The ultimate goal of cannabis cryopreservation is the long-term preservation of elite genetics. Theoretically, clonal propagation through plant tissue culture should produce true to type plants, but in many cases can lead to a higher rate of somatic mutations, a phenomenon known as somaclonal variation [14]. While somaclonal variation has not been reported in *Cannabis* and previous work has reported that there were no mutations detected in micropropagated plants [13], this study used low resolution ISSR markers that would miss many potential mutations. A more recent study using whole genome sequencing identified significant genetic variation within a single cannabis plant [11], suggesting that cannabis is prone to accumulating mutations. This stresses the potential value of cryopreservation to maintain cannabis genetics, but also highlights the need to ensure that the process results in true-to-type plants.

In this study, cryopreserved plants (Cultivar 1) were grown to maturity along with both control groups to compare the morphological and chemical characteristics. Overall, there were no significant differences in any of the gross morphological characteristics among the treatments, including total plant fresh weight, fresh or dry inflorescence weight, or fresh or dry trim weight (Table 3 and Table 4). Likewise, there were no significant differences in total cannabinoid content or total terpene content among the treatments (Table 5 and Table 6). This is in agreement with Lata et al., 2019 [19], who found that cryopreserved axillary buds resulted in plants with similar THC and CBD levels as the parent material.

However, in the present study there were differences in some of the minor cannabinoids in both the flower and the trim (Table 5). For example, the control plants produced 0.14% CBG, whereas the cryopreserved plants did not produce any detectable amount. Likewise, this was also observed for some of the minor terpenes such as α-pinene, which was present in the controls but not detected in the cryopreserved plants (Table 6). Unfortunately, this was only done with a single genotype and these minor compounds were not evaluated in previous studies, so it is difficult to determine if this was an anomaly or a reproducible difference. While the cause for discrepancies in the minor compounds is not known, it is important that there were no differences in any of the major compounds. Furthermore, while the total cannabinoid and terpene contents were not significantly different among treatments, they were numerically higher in the cryopreserved plants, showing that the differences in minor cannabinoids and terpenes were not a result of lower overall biosynthesis. While further work is warranted to investigate this peculiarity and compare cryopreserved plants at a genetic level, these results demonstrate that plants produced from cryopreserved nodal explants are largely true-to-type.

## 4. Conclusions

This study includes a series of experiments to establish an efficient cryopreservation system for *Cannabis sativa*. Based on these results, it was determined that standard vitrification-based cryopreservation can be effectively used to cryopreserve nodal explants from in vitro plants, representing an efficient and sanitary protocol for long-term germplasm conservation. While the protocol worked across all 13 genotypes evaluated, there was significant variation in both survival and regrowth. While further optimization of the cryopreservation protocol may improve outcomes, it is likely that it results from variable responses to in vitro culture in general and it may be more effective to address the basic culture system to improve the results rather than further refining the cryopreservation methods. Regardless, the final protocol was successful in all 13 commercial genotypes tested and could be used for long-term preservation.

## Figures and Tables

**Table 1:** Survival and regrowth of Cultivar 1 explants from different freezing methods and incubation on various recovery media.

Cryopreservation Treatment	Freezing Method	Recovery Media	Survival (%)	Regrowth (%)
Control	N/A	HalfSMM	100 ± 0	90 ± 10 abc
	N/A	HalfSMM + GA3	100 ± 0	96.7 ± 3.3 a
	N/A	MSbasal	100 ± 0	95 ± 2.9 ab
	N/A	MSbasal + GA3	100 ± 0	100 ± 0 a
	N/A	SMM	100 ± 0	93.3 ± 3.3 ab
no LN2	Droplet Vitrification	HalfSMM	76.7 ± 10.9	60 ± 12.6 cdef
	Droplet Vitrification	HalfSMM + GA3	66.7 ± 9.9	50 ± 13.4 def
	Droplet Vitrification	MSbasal	75 ± 5	35 ± 9.6 defg
	Cryogenic vial	MSbasal	80 ± 11.5	50 ± 17.3
	Droplet Vitrification	MSbasal + GA3	85 ± 9.6	75 ± 5 cdef
	Cryogenic vial	MSbasal + GA3	55 ± 9.6	55 ± 9.6
	Droplet Vitrification	SMM	85 ± 9.6	69.2 ± 15 abcd
	Cryogenic vial	SMM	80 ± 8.2	55 ± 12.6
LN2	Droplet Vitrification	HalfSMM	33.3 ± 6.7	20 ± 0 fg
	Droplet Vitrification	HalfSMM + GA3	43.3 ± 6.1	23.3 ± 3.3 fg
	Droplet Vitrification	MSbasal	50 ± 5.8	30 ± 12.9 efg
	Cryogenic vial	MSbasal	55 ± 20.6	10 ± 10
	Droplet Vitrification	MSbasal + GA3	30 ± 5.8	15 ± 15 fg
	Cryogenic vial	MSbasal + GA3	30 ± 5.8	20 ± 8.2
	Droplet Vitrification	SMM	30 ± 11.3	3.3 ± 3.3 g
	Cryogenic vial	SMM	65 ± 5	15 ± 5

Survival and recovery were analyzed separately. Significant data is followed by different lower-case letters (*p* < 0.05). Significant differences were only found between cryopreservation treatments and media interactions when the droplet-vitrification method was used.

**Table 2:** Survival and regrowth of 13 genotypes of cryopreserved *Cannabis sativa*. Conventional vitrification-based cryopreservation was conducted using nodal explants from in vitro plantlets and includes control explants with not treatment (control), explants that went through the entire protocol except for freezing (no LN2), and explants that were cryopreserved in liquid nitrogen (LN2).

Genotype	Treatment					
	Control		No LN		LN	
	Survival (%)	Regrowth (%)	Survival (%)	Regrowth (%)	Survival (%)	Regrowth (%)
Cultivar 1	100 ± 0	100 ± 0 a	95 ± 5	85 ± 5 abcde	80 ± 10	60 ± 10 bcdefg
Cultivar 2	100 ± 0	93.3 ± 3.3 ab	100 ± 0	90 ± 10 abc	80 ± 5.8	66.7 ± 3.3 abcdefg
Cultivar 3	100 ± 0	95 ± 5 a	95 ± 5	85 ± 5 abcde	70 ± 0	35 ± 15 efg
Cultivar 4	100 ± 0	100 ± 0 a	90 ± 0	90 ± 0 abcde	60 ± 0	45 ± 5 defg
Cultivar 5	86.7 ± 11.6	37.4 ± 14.1 efg	83.3 ± 5.8	66.7 ± 5.8 abcdefg	60 ± 26.5	43.3 ± 25.2 cdefg
Cultivar 6	76.7 ± 15.3	30 ± 25.2 fg	80 ± 5.8	70 ± 5.8 abcdefg	63.3 ± 15.3	50 ± 10 bcdefg
Cultivar 7	93.3 ± 5.8	83.3 ± 5.8 abcd	83.3 ± 11.6	70 ± 10 abcdefg	43.3 ± 15.3	30 ± 10 fg
Cultivar 8	100 ± 0	96.7 ± 3.3 a	80 ± 11.6	70 ± 5.8 abcdefg	60 ± 10	43.3 ± 8.8 cdefg
Cultivar 9	93.3 ± 6.7	76.7 ± 6.7 bcde	86.7 ± 6.7	66.7 ± 8.8 abcdefg	50 ± 15.3	26.7 ± 12 g
Cultivar 10	93.3 ± 3.3	76.7 ± 8.8 bcde	86.7 ± 8.8	60 ± 5.8 abcdefg	73.3 ± 8.8	26.7 ± 8.8 g
Cultivar 11	86.7 ± 13.3	73.3 ± 17.6 abcdef	80 ± 5.8	70 ± 10 abcdefg	80 ± 10	50 ± 5.8 bcdefg
Cultivar 12	99.3 ± 3.3	70 ± 5.8 abcdefg	70 ± 0	66.6 ± 3.3 abcdefg	66.7 ± 8.8	43.3 ± 8.8 cdefg
Cultivar 13	100 ± 0	93.3 ± 6.7 ab	90 ± 5.8	43.3 ± 8.8 cdefg	66.7 ± 8.8	46.7 ± 6.7 cdefg

Survival and regrowth were analyzed separately. Means and standard errors followed by different lower-case letters indicate significant differences (*p* < 0.05).

**Table 3:** Cultivar 1 fresh weight harvest data from ex vitro cultivation.

Cryopreservation Treatment	Whole Plant Fresh Weight (g)	Stem Fresh Weight (g)	Trim Fresh Weight (g)	Bud Fresh Weight (g)
Control	3007.7 ± 101.7	196.5 ± 20	272 ± 45.6	258 ± 39.9
no LN2	3033.5 ± 216.5	173 ± 31	306 ± 96	273.5 ± 89.5
LN2	3108 ± 296	186.5 ± 50.5	332.5 ± 163.5	308 ± 82

**Table 4:** Cultivar 1 dry weight, moisture content, and yield data from ex vitro cultivation.

Cryopreservation Treatment	Trim Dry Weight (g)	Trim Moisture Content (g)	Bud Dry Weight (g)	Bud Moisture Content (%)	Dry Bud Yield per Plant (g/g)
Control	46.1 ± 3.8	81.9 ± 3.6	54 ± 8.9	79.1 ± 0.3	0.018 ± 0.002
no LN2	45.3 ± 12.5	82.2 ± 9.7	51 ± 13	80.9 ± 1.2	0.017 ± 0.003
LN2	34.6 ± 1.3	86.1 ± 7.2	63.3 ± 16.3	79.4 ± 0.2	0.02 ± 0.003

**Table 5:** Cannabinoid content from ex vitro cultivation of Cultivar 1 treatments.

	Bud			Trim		
	Control	no LN2	LN2	Control	no LN2	LN2
CBDV	0.13 ± 0.02 a	0.045 ± 0.05 b	0 b	0.01 ± 0.006 a	0.01 ± 0.01 a	0 a
CBC	0.025 ± 0.02 a	0 a	0 a	0 a	0 a	0 a
d8THC	0.025 ± 0.003 a	0.01 ± 0 b	0.01 ± 0 ab	0 a	0 a	0 a
CBG	0.14 ± 0.01 a	0.06 ± 0.06 b	0 b	0.0032 ± 0.003 a	0.015 ± 0.02 b	0 b
CBGA	0.33 ± 0.03 a	0.2 ± 0.1 b	0.065 ± 0.005 b	0.095 ± 0.01 a	0.04 ± 0.04 b	0.005 ± 0.005 b
CBD	0 b	0.065 ± 0.07 a	0.14 ± 0.02 a	0 a	0 a	0 a
CBDA	0.07 ± 0.004 a	0.03 ± 0.03 b	0.01 ± 0.01 b	0.018 ± 0.003 a	0.01 ± 0.01 ab	0 b
d9THC	0.73 ± 0.09 a	0.68 ± 0.1 a	0.55 ± 0.005 a	0.29 ± 0.01 a	0.22 ± 0.04 b	0.15 ± 0.01 b
d9THCA	11.18 ± 0.9 a	11.1 ± 1.2 a	12.7 ± 0.9 a	3.67 ± 0.3 a	2.83 ± 0.5 b	2.97 ± 0.4 ab
Total	12.54 ± 1 a	12.14 ± 0.9 a	13.49 ± 1 a	4.27 ± 0.4 a	3.11 ± 0.6 b	3.13 ± 0.4 ab

Values are presented as *%w/w* of the original dried material. Means followed by the same letter are not significantly different (*p* < 0.05) according to Tukey’s HSD mean separation test.

**Table 6:** Terpene content from Cultivar 1 ex vitro cultivation. Values are presented as %w/w of the original dried material.

	Bud			Trim		
	Control	no LN2	LN2	Control	no LN2	LN2
α-pinene	0.073 ± 0.005	0.048 ± 0.01	0	0.025 ± 0.001	0.027 ± 0.004	0
β-pinene	0.083 ± 0.006	0.056 ± 0	0.076 ± 0	0.029 ± 0	0.03 ± 0	0.033 ± 0
Myrcene	0.34 ± 0.04	0.25 ± 0.08	0.41 ± 0.2	0	0	0
Carene	0.019 ± 0.002	0.013 ± 0.004	0	0.006 ± 0.0003	0.0067 ± 0.002	0
α-terpinene	0.026 ± 0.002	0.014 ± 0.005	0.021 ± 0.01	0.0063 ± 0.0004	0.0066 ± 0.002	0.0065 ± 0.003
ρ-cymene	0.011 ± 0	0.016 ± 0.004	0.023 ± 0.01	0	0	0
Limonene	0.013 ± 0.001	0.011 ± 0.0004	0.016 ± 0.007	0.0051 ± 0.0004	0.0071 ± 0.001	0
Ocimene	0.038 ± 0.004	0.033 ± 0.008	0.051 ± 0.02	0.017 ± 0.001	0.022 ± 0.004	0.02 ± 0.002
δ-terpinene	0.018 ± 0.001	0.01 ± 0.003	0.014 ± 0.006	0.0087 ± 0.0003	0.0048 ± 0.001	0.0045 ± 0.002
Terpinolene	0.25 ± 0.03	0.16 ± 0.05	0.25 ± 0.1	0.06 ± 0.005	0.065 ± 0.02	0.065 ± 0.02
Linalool	0.02 ± 0.002	0.013 ± 0.004	0.018 ± 0.007	0.0087 ± 0.0007	0.0099 ± 0.0007	0.01 ± 0.002
Osipulegol	0.011 ± 0.001	0.0073 ± 0.001	0.012 ± 0.005	0.0041 ± 0.0004	0.0047 ± 0.001	0.0033 ± 0.003
β-caryophyllene	0.07 ± 0.003	0.063 ± 0.01	0.071 ± 0.03	0.076 ± 0.004	0.097 ± 0.01	0.09 ± 0.001
α-humulene	0.014 ± 0.0006	0.014 ± 0.003	0.016 ± 0.006	0.017 ± 0.0009	0.022 ± 0.002	0.02 ± 0.0006
Nerolidol 2	0.6 ± 0.04	0.46 ± 0.09	0.52 ± 0.2	0.47 ± 0.03	0.53 ± 0.07	0.49 ± 0.2
Guaiol	0.014 ± 0.01	0.025 ± 0.02	0.057 ± 0.008	0	0.025 ± 0.03	0.03 ± 0.03
α-terpineol	0.048 ± 0.004	0.037 ± 0.009	0.044 ± 001	0.021 ± 0.002	0.023 ± 0.004	0.014 ± 0.01
Total	1.64 ± 0.1	1.23 ± 0.3	1.69 ± 0.7	0.83 ± 0.05	0.933 ± 0.2	0.92 ± 0.3

## Data Availability

All data, tables, and figures in this manuscript are original. Some of the data in this study was presented at the Society for In Vitro Biology Conference in 2019.

## Supplementary Materials

The following are available online at https://www.mdpi.com/article/10.3390/plants10091794/s1, Table S1: Historical major cannabinoid profiles of *C. sativa* cultivars used in this study. Figure S1: HPLC-DAD chromatographs of ex vitro Cultivar 1 bud and trim material: (A,C,E,G) control bud samples; (B,D,F,H) control trim samples; (I,K) no LN2 bud samples; (J,L) no LN2 trim samples; (M,O) LN2 bud samples; (N,P) LN2 trim samples. Figure S2: GC-FID chromatographs of ex vitro Cultivar 1 bud and trim material: (A,C,E,G) control bud samples; (B,D,F,H) control trim samples; I,K) no LN2 bud samples; (J,L) no LN2 trim samples; (M,O) LN2 bud samples; (N,P) LN2 trim samples.
