# Supplementary material for: Cryopreservation of 13 Commercial Cannabis sativa Genotypes Using In Vitro Nodal Explants
Source: Plants (Basel). 2021 Aug 28;10(9):1794. doi: 10.3390/plants10091794 (PMC8470898; doi:10.3390/plants10091794)
Supplement: Supplementary file 1 [file plants-10-01794-s001.zip › Table S1.pdf]

Supplemental Table S1: Historical major cannabinoid profiles of *C. sativa* strains used in this study.

**Strain**	**Historic THC content (%w/w)**	**Historic CBD content (%w/w)**
Strain 1	20-25	<0.1
Strain 2	6.8-9.2	7.65-10.4
Strain 3	<1	12.6-15.4
Strain 4	17-27	<0.1
Strain 5	20-25	<0.1
Strain 6	19-24	0.04 to <0.1
Strain 7	6-10	6-10
Strain 8	15-25	<0.1
Strain 9	8	4.9
Strain 10	22-27	0.04 to <0.1
Strain 11	23-28	<0.1
Strain 12	<0.1	19.8
Strain 13	15-20	<0.1
