# Supplementary figures and images for: Cryopreservation of 13 Commercial Cannabis sativa Genotypes Using In Vitro Nodal Explants
Source: Plants (Basel). 2021 Aug 28;10(9):1794. doi: 10.3390/plants10091794 (PMC8470898; doi:10.3390/plants10091794)

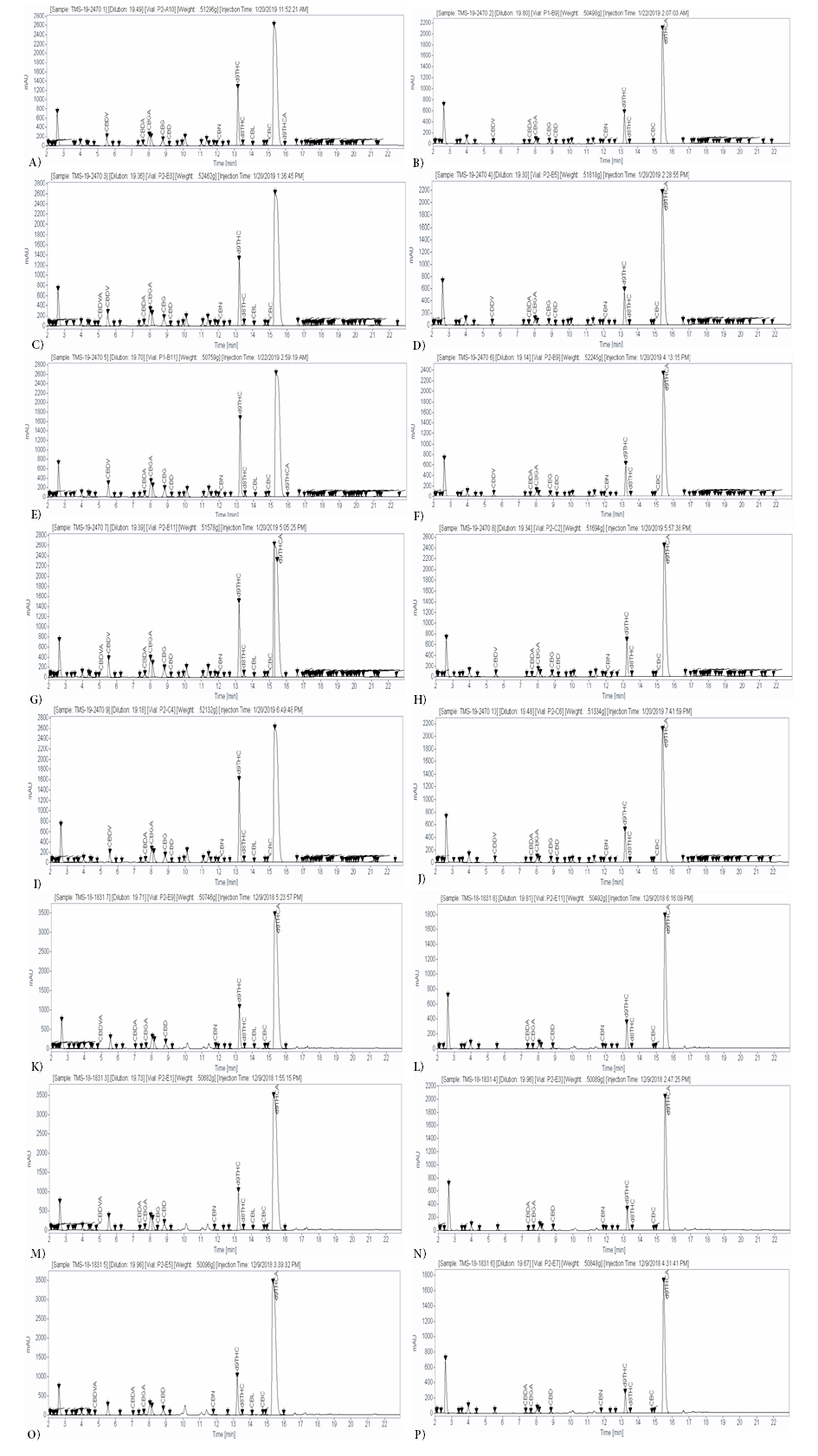

Supplement: Supplementary file 1 [file plants-10-01794-s001.zip › Supplemental Figure S1.PNG]

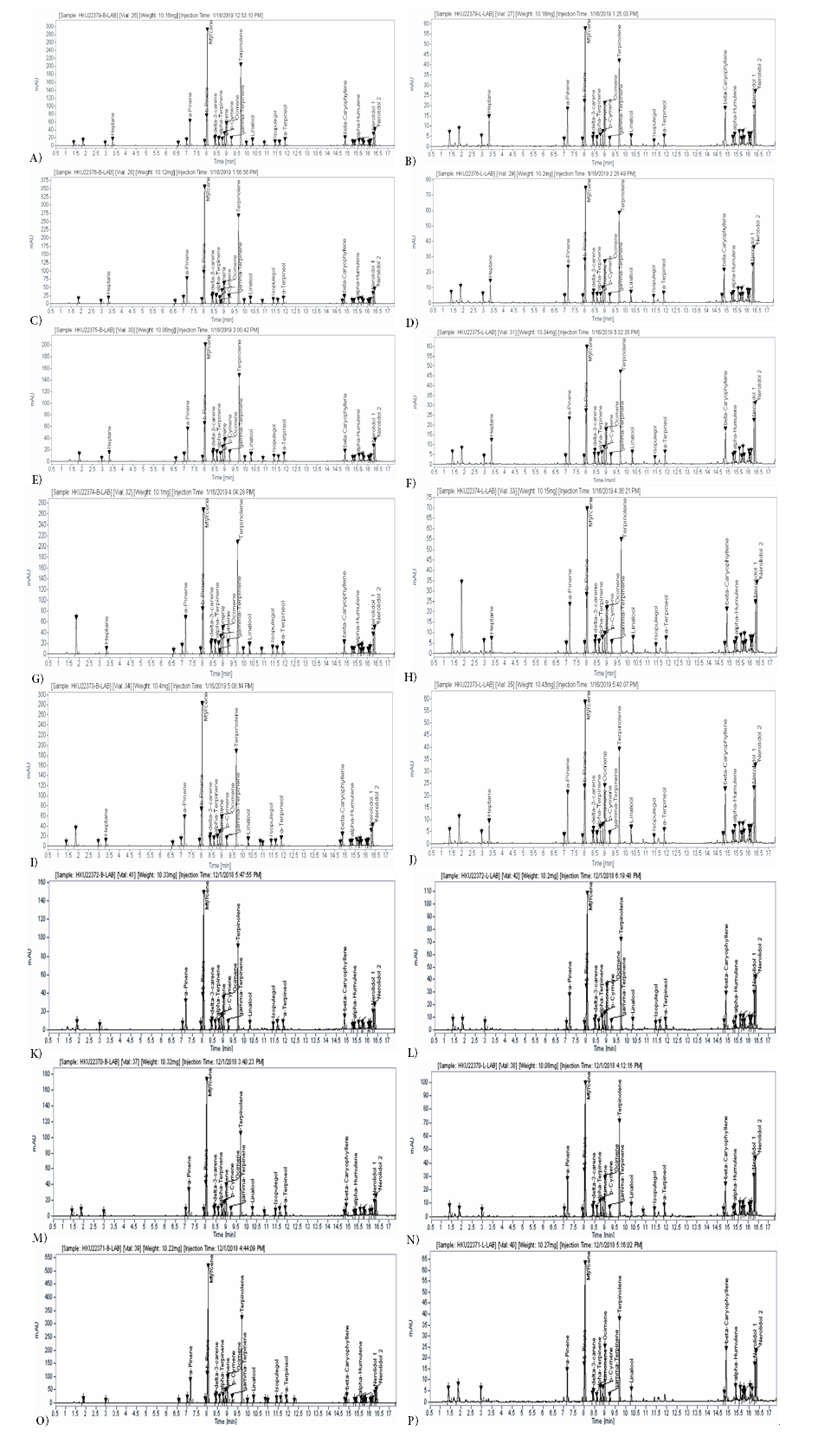

Supplement: Supplementary file 1 [file plants-10-01794-s001.zip › Supplemental Figure S2.PNG]
